# Supplementary material for: Optimising network modelling methods for fMRI
Source: Neuroimage. 2020 May 1;211:116604. doi: 10.1016/j.neuroimage.2020.116604 (PMC7086233; doi:10.1016/j.neuroimage.2020.116604)
Supplement: Multimedia component 1 [file mmc1.pdf]

Appendix A. Inline Supplementary Figures

BRAINNET CNN

Parameters	Fine-tuned Values
Edge2Edge	256
Edge2Node	128
Node2Graph	256
Fully Connected-1,2,3	256,128, 30
Loss	mean_squared_error/ binary_crossentropy
Optimizer	SGD
Batch Size	4
Learning Rate	0.0000001

2D RCNN

Parameters	Fine-tuned Values
Recurrent Blocks	2-4
Residual Blocks	4
Kernel size	3
Number of kernels	128
Fully Connected-1,2	128, 64
Loss	mean_squared_error/ binary_crossentropy
Optimizer	SGD
Batch Size	128
Learning Rate	0.0001

Table A.4: The tuned hyper-parameters for BrainNetCNN and 2DRCNN.

**Method**	**r value**
No Deconfouding	0.2934
Deconfounding Age	0.2815
Deconfounding Age + Sex	0.2720
Deconfounding Sex	0.2714
Deconfounding ethnicity	0.2919
Deconfounding Height	0.2739
**Deconfounding rfMRI motion**	0.2552
**Deconfounding FS IntraCranial Volume**	0.2240
**Deconfounding FS BrainSeg Volume**	0.2378
Deconfounding all variables	0.1926

Table A.5: The impact of deconfounding each confound separately on the prediction of fluid intelligence in the HCP data.

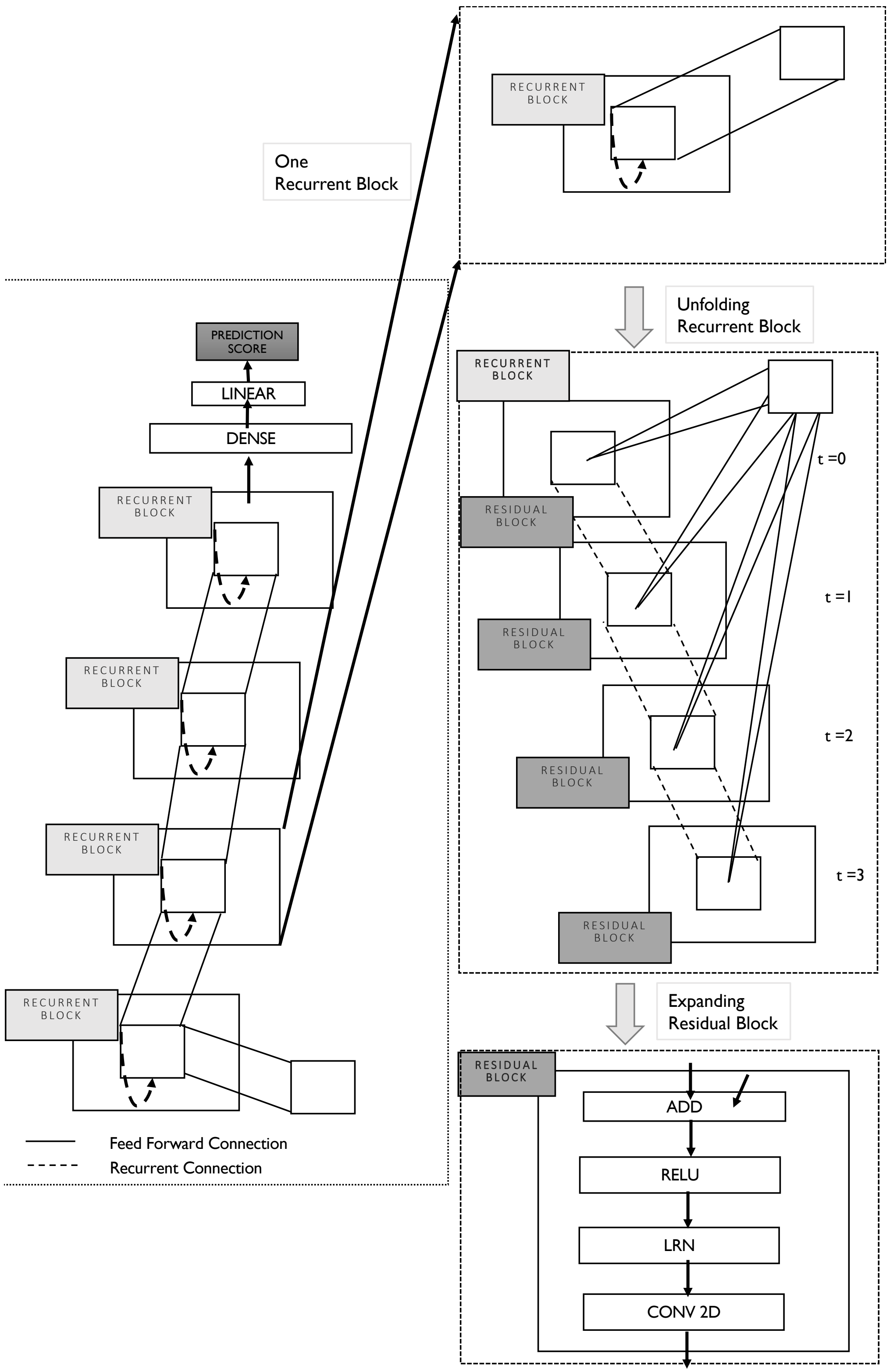

Figure A.12: The proposed architecture of 2D RCNN. A recurrent block is unfolded on the right for t = 3 time steps. At t = 0, it is only a feed-forward network with a single residual block. At t =3, it has depth of 4 with additional recurrent connections. Each residual block is further composed of addition, batch normalization, convolution and activation layers.

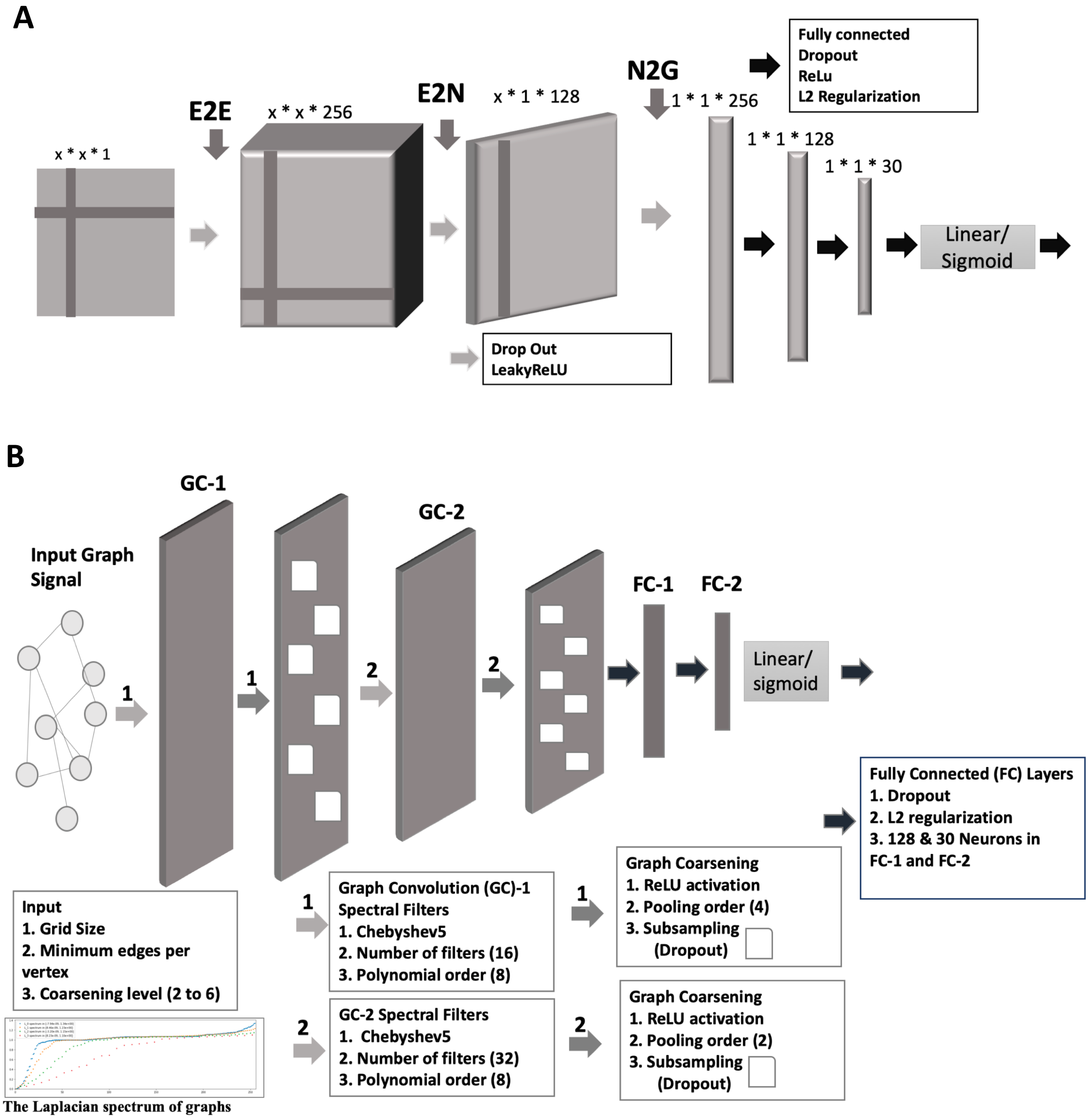

Figure A.13: The architectures of DNNs: **(A)** The BrainNetCNN architecture, where the E2E layer considers weights of all neighbouring edges (adjacent brain regions), the E2N layer convolves netmats with 1D convolutional filter producing a single output for each node and finally the N2G layer reduces dimensionality. **(B)** the schematic of GraphCNN, which can be summarized as $H^{(l+1)} = [\tilde{D}^{(-1/2)}\tilde{A}\tilde{D}^{(-1/2)}H^{(l)}W^{(l)}]$, where A is the adjacency matrix, W is weight matrix and D is degree matrix. Input graph signals pass through a set of convolution, pooling and fully connected layers resulting in producing a single output score corresponding to non-imaging variable for each subject's functional connectivity matrix.

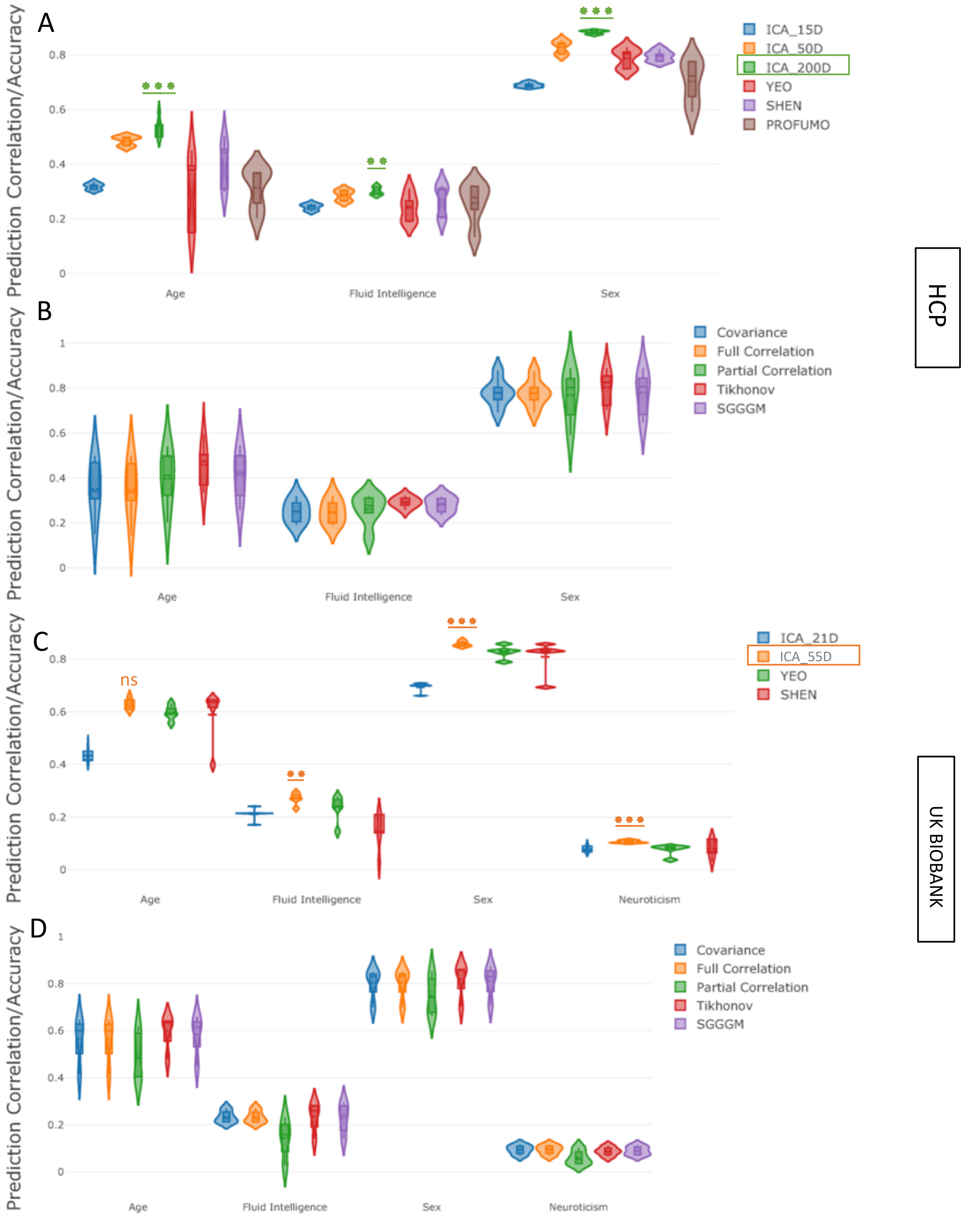

Figure A.14: The impact of various parcellation strategies and functional connectivity estimation methods on prediction power for non-imaging variables **without deconfounding**. **[A,B] (HCP Data), [C,D] (UKB Data):** The violin plots in **A** and **C** show the prediction variability over 5 measures of functional connectivity estimates and in **B** and **D** show the prediction variability the over different parcellation schemes. For HCP, the ICA based parcellation schemes are ICA_15D, ICA_50D and ICA_200D, and for UKB are ICA_21D and ICA_55D, where D = the number of parcels. For both HCP and UKB, SHEN parcellation was 268D, YEO was 100D, and PROFUMO was 50D (for HCP only). The stars refer to comparison against the next-best method.

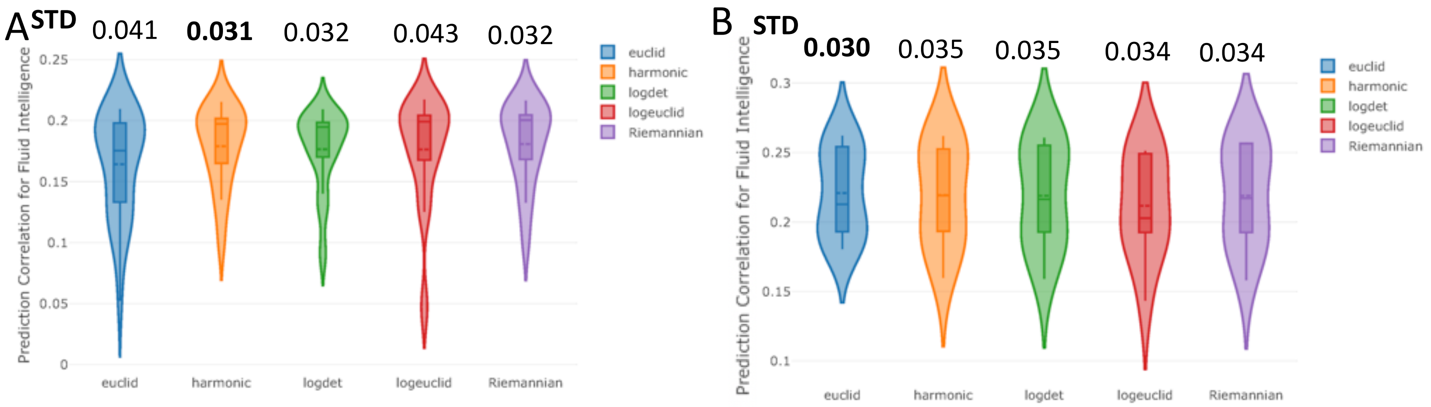

Figure A.15: The impact of various reference mean estimation techniques during tangent space parameterization on prediction of non-imaging variables **after deconfounding**. [A] **(HCP Data), [B] (UKB Data)** These figures show the prediction correlation for fluid intelligence scores when functional connectivity estimates are projected into tangent space, using different reference means. The violin plots represent the prediction variability over 4 different parcellation schemes and 5 measures of functional connectivity estimates in the tangent space.

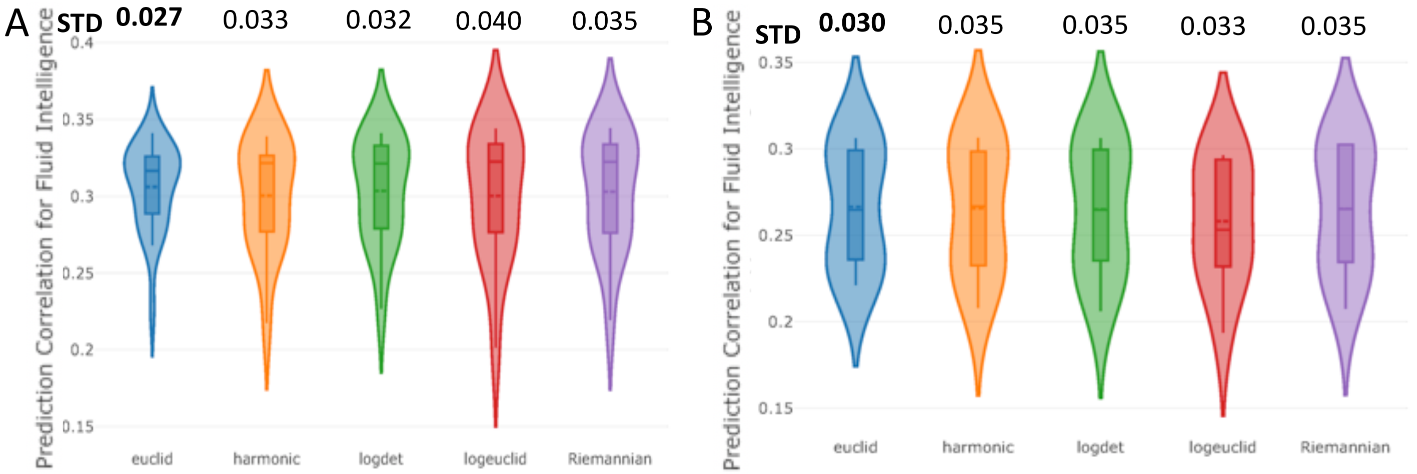

Figure A.16: The impact of various reference mean estimation techniques during tangent space parameterization on prediction of non-imaging variables **without deconfounding**. [A] **(HCP Data), [B] (UKB Data)** These figures show the prediction correlation for fluid intelligence scores when functional connectivity estimates are projected into tangent space, using different reference means. The violin plot represents the prediction variability over 3 different parcellation schemes and 5 measures of functional connectivity estimates in the tangent space.

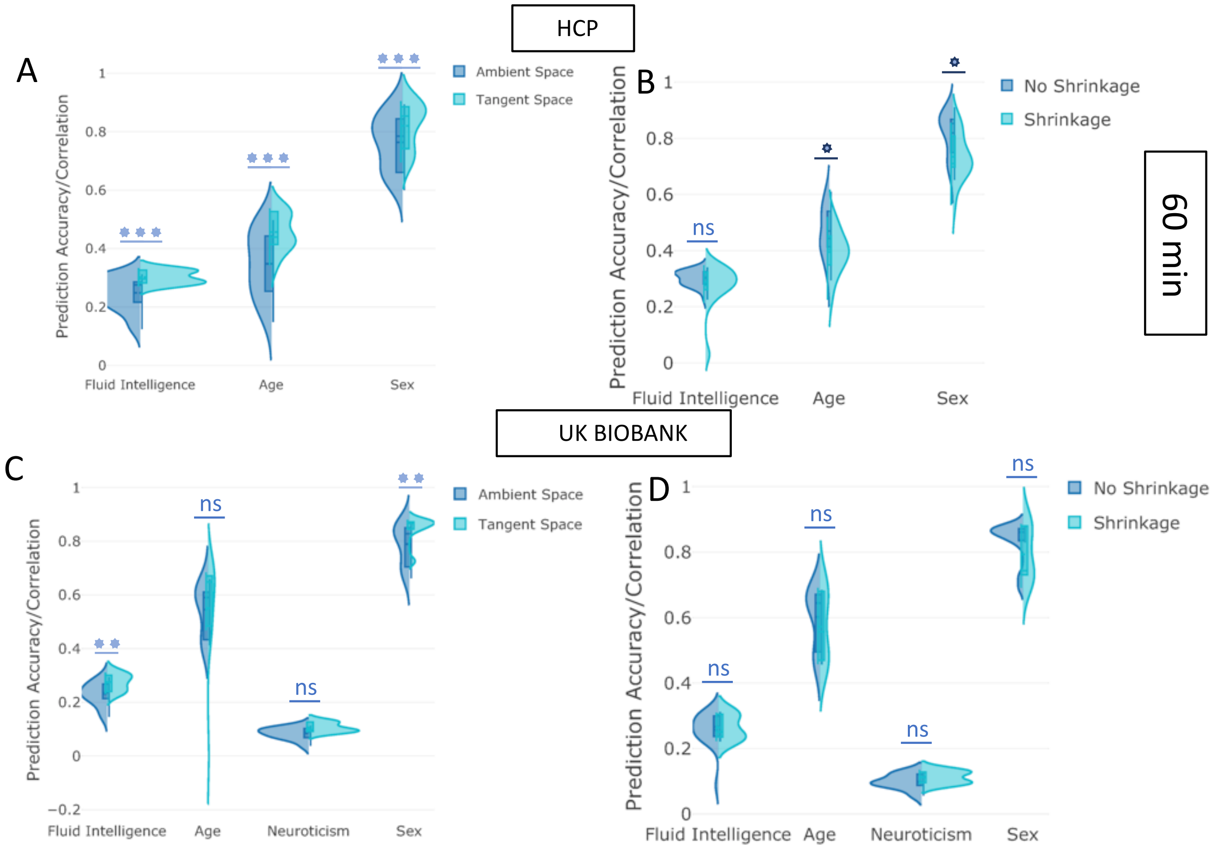

Figure A.17: The impact of projecting to tangent space and applying shrinkage in tangent space on prediction power **before deconfounding**. [A-B] **(HCP Data), [C,D] (UKB):** The y-axis depicts the prediction accuracy/correlation for different behavioural measures. "Tangent Space" means that tangent space projection was applied on functional connectivity estimates (originally in the "Ambient Space"). The "Shrinkage" strategy means that non-isotropic PoSCE shrinkage was applied to connectivity estimates in tangent space before feeding to the predictor/classifier. "No Shrinkage" means that projected functional connectivity estimates in tangent space were directly fed to the predictor/classifier, and did not undergo PoSCE shrinkage. The violin plots show the prediction variability over 4 different parcellation schemes and 5 measures of functional connectivity estimates.

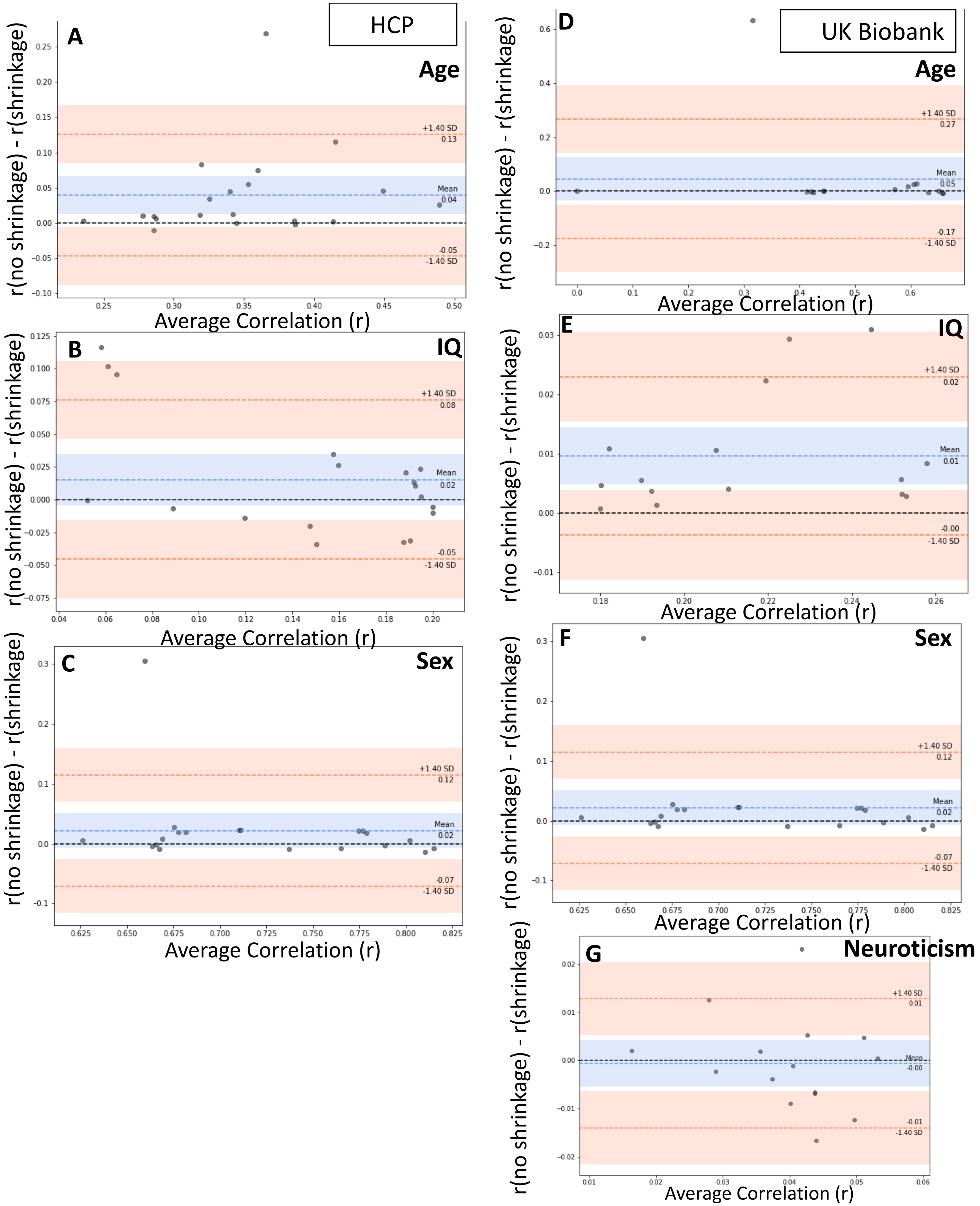

Figure A.18: The impact of applying shrinkage in tangent space on prediction power **after deconfounding**. **[A,B,C] (HCP Data), [D,E,F,G] (UKB Data):** The y-axis depicts the difference of two methods (no shrinkage - shrinkage) and x-axis depicts the mean of methods ((no shrinkage + shrinkage) /2).

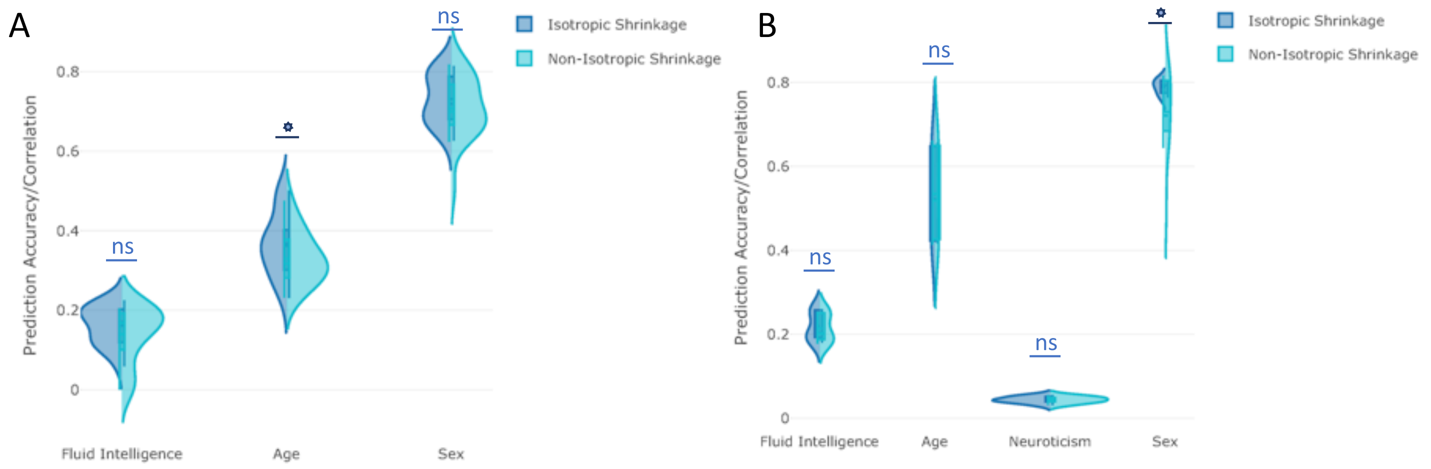

Figure A.19: The impact of isotropic versus non-isotropic shrinkage in tangent space on prediction power for non-imaging variables **after deconfounding**. [**A**] **(HCP Data), [B](UKB)**: The y-axis depicts the prediction accuracy/correlation for different non-imaging measures. Isotropic Shrinkage means that Ledoit-Wolf shrinkage was applied to projected functional connectivity estimates in tangent space before they were fed to a predictor/classifier. The Non-Isotropic Shrinkage strategy means that PoSCE shrinkage was applied to connectivity estimates in tangent space before they were fed to a predictor/classifier.

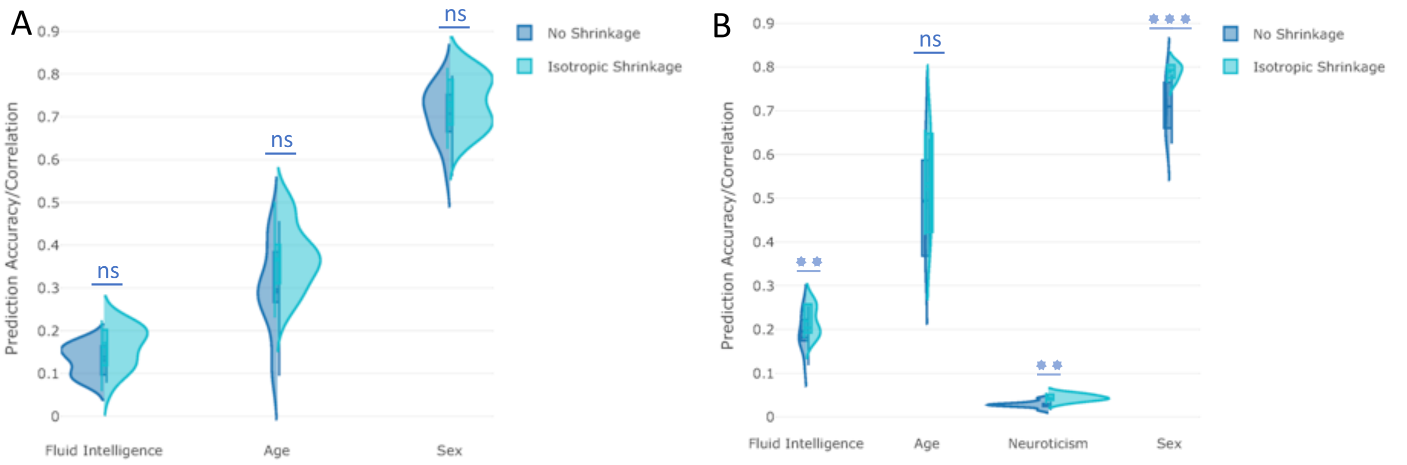

Figure A.20: The impact of applying isotropic shrinkage in tangent space on prediction power after deconfounding. [**A**] **(HCP Data), [B](UKB)**: The y-axis depicts the prediction accuracy/correlation for different non-imaging measures. Isotropic Shrinkage means that Ledoit-Wolf shrinkage was applied to projected functional connectivity estimates in tangent space before they were fed to a predictor/classifier. "No Shrinkage" means that projected functional connectivity estimates in tangent space were directly fed to the predictor/classifier, and did not undergo Ledoit-Wolf shrinkage.

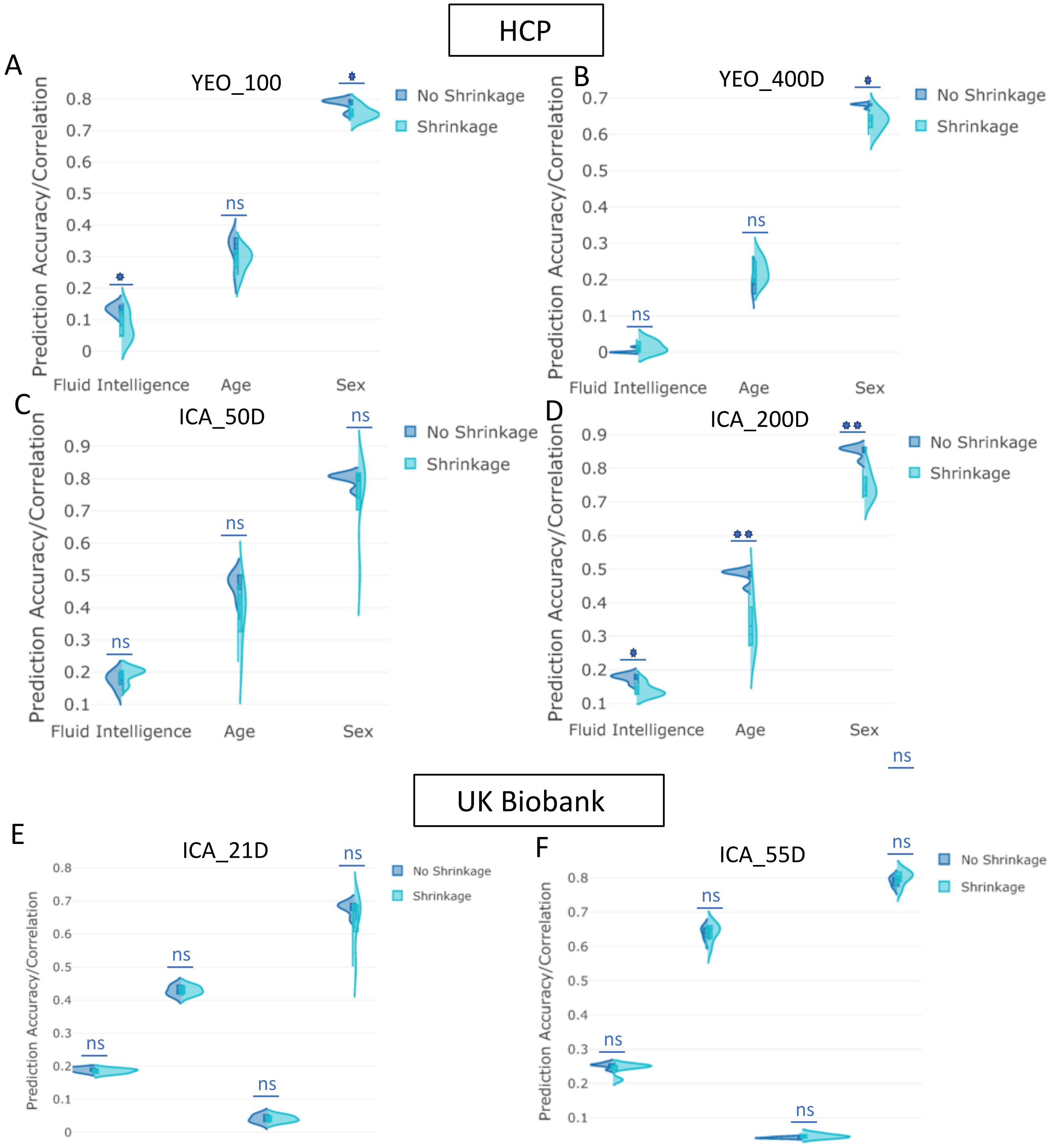

Figure A.21: The impact of applying shrinkage in tangent space on prediction power varying the number of parcels. **[A-D] (HCP Data), [E,F](UKB)**: The y-axis depicts the prediction accuracy/correlation for different behavioural measures. The "Shrinkage" strategy means that non-isotropic PoSCE shrinkage was applied to connectivity estimates in tangent space before feeding to the predictor/classifier. "No Shrinkage" means that projected functional connectivity estimates in tangent space were directly fed to the predictor/classifier, and did not undergo PoSCE shrinkage. The "YEO_400D" means that 400D YEO parcellation was applied. The violin plots show the prediction variability over 5 measures of functional connectivity estimates for the mentioned parcellation scheme (e.g., "YEO_400D")

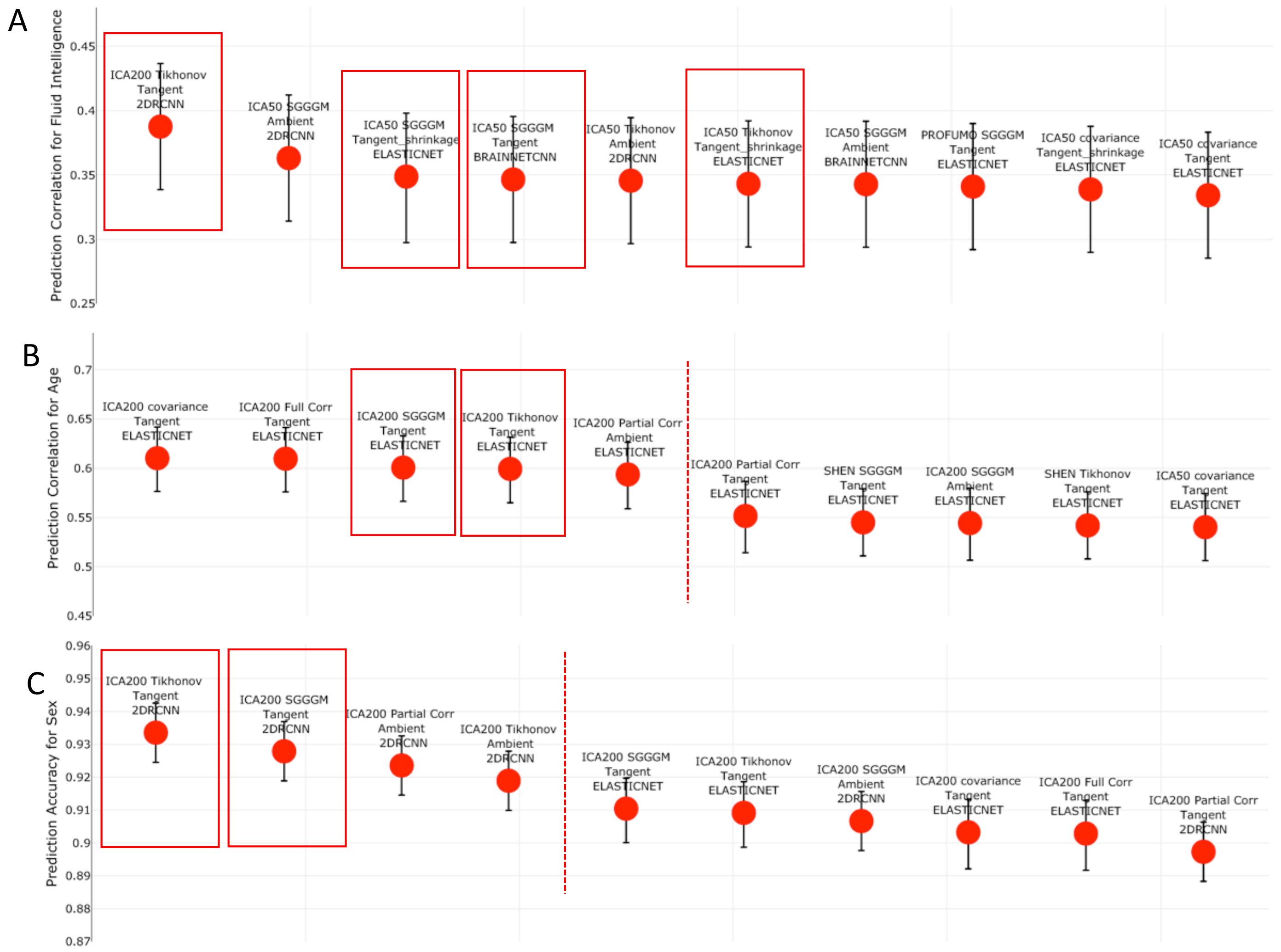

Figure A.22: The top performing ten configurations for the prediction of each non-imaging variable by dataset **without deconfounding**. **[A,B,C] (HCP Data)** Each data point represents a different configuration strategy that may vary in terms of parcellation strategy, the functional connectivity estimation method, whether tangent space parameterization was employed, whether tangent space regularization was employed, and the predictor/classifier that was used. The first word indicates the parcellation strategy, and the second word refers to the functional connectivity estimation method. The third word refers to the geometry in which classifier is applied, ambient referring to non-tangent space and tangent referring to the projected covariance matrices in tangent space. If non-isotropic shrinkage was applied after projecting covariance matrices to tangent space, the fourth word will be "shrinkage". The last word indicates the type of classifier/predictor that was used. The highlighted red blocks show the recommended pipelines (rationale explained in Section 5), and red dotted lines highlight the point when the error bar of pipeline after the dotted line is out of range from the error bar of the top (first) pipeline.

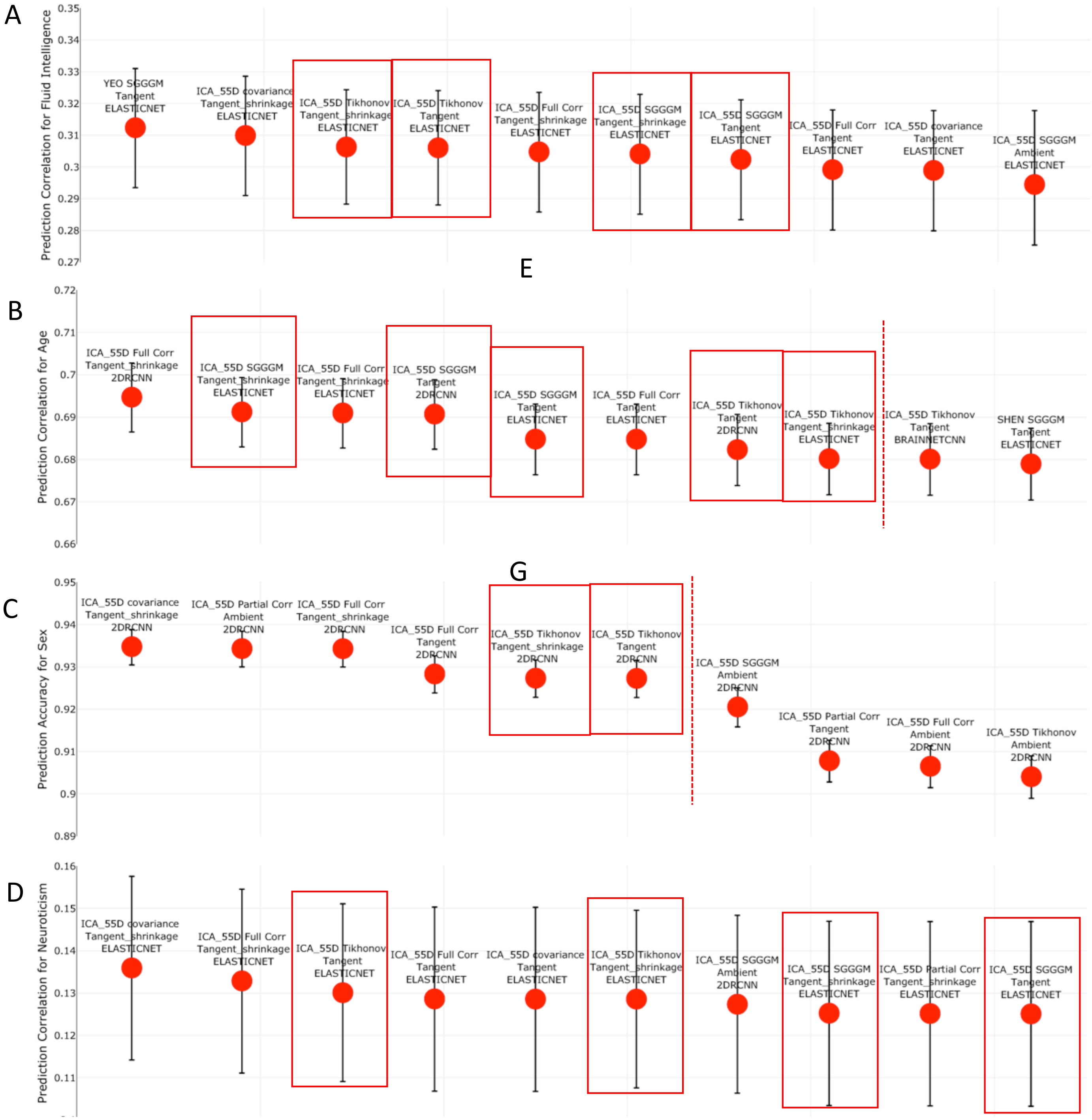

Figure A.23: The top performing ten configurations for the prediction of each non-imaging variable by dataset **without deconfounding**. **[A,B,C,D] (UKB Data)** Each data point represents a different configuration strategy that may vary in terms of parcellation strategy, the functional connectivity estimation method, whether tangent space parameterization was employed, whether tangent space regularization was employed, and the predictor/classifier that was used. The first word indicates the parcellation strategy, and the second word refers to the functional connectivity estimation method. The third word refers to the geometry in which classifier/predictor is applied, ambient referring to non-tangent space and tangent referring to the projected covariance matrices in tangent space. If non-isotropic shrinkage was applied after projecting covariance matrices to tangent space, the fourth word will be "shrinkage". The last word indicates the type of classifier/predictor that was used. The highlighted red blocks show the recommended pipelines (rationale explained in Section 5), and red dotted lines highlight the point when the error bar of pipeline after the dotted line is out of range from the error bar of the top (first) pipeline.

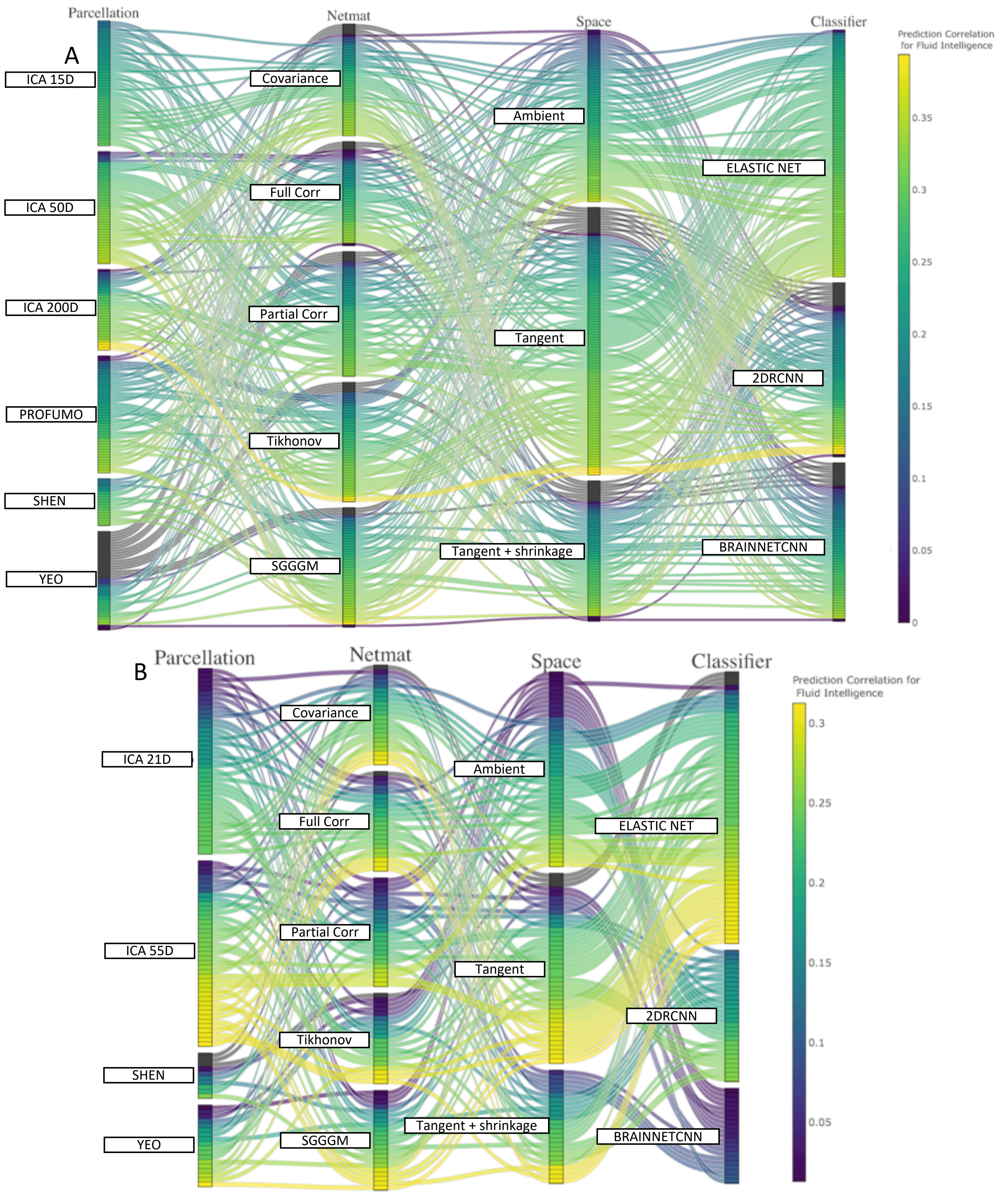

Figure A.24: This parallel coordinates plot provides a visualization of all possible combinations of options in the pipeline to predict fluid intelligence scores from functional connectivity **without deconfounding**. **[A] (HCP Data), [B] (UKB Data):** The lines are color-coded according to their prediction performance.

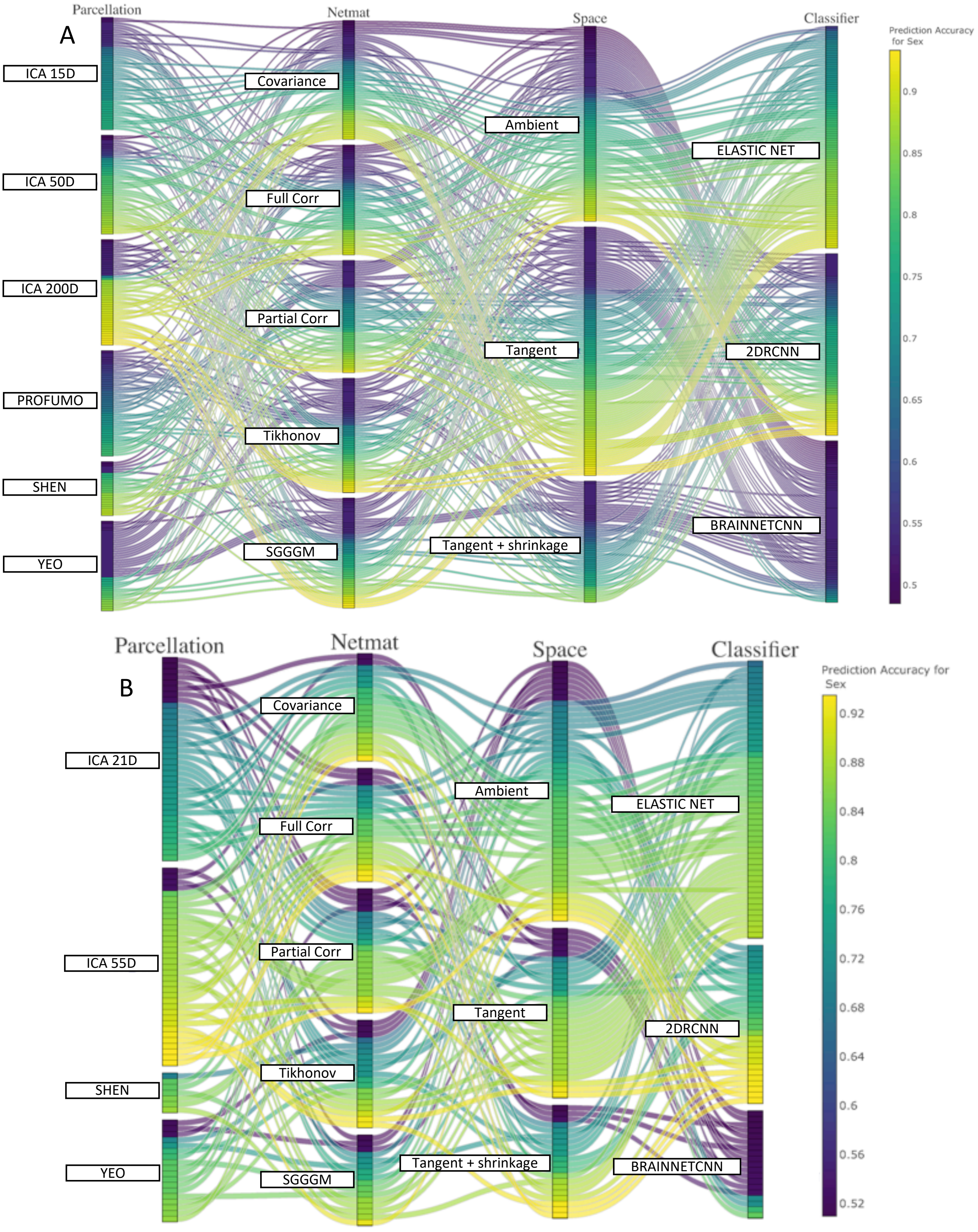

Figure A.25: This parallel coordinates plot provides a visualization of all possible combinations of options in the pipeline to pipeline to predict sex from functional connectivity **without deconfounding**. [A] **(HCP Data),** [B] **(UKB Data):** The lines are color-coded according to their prediction performance.

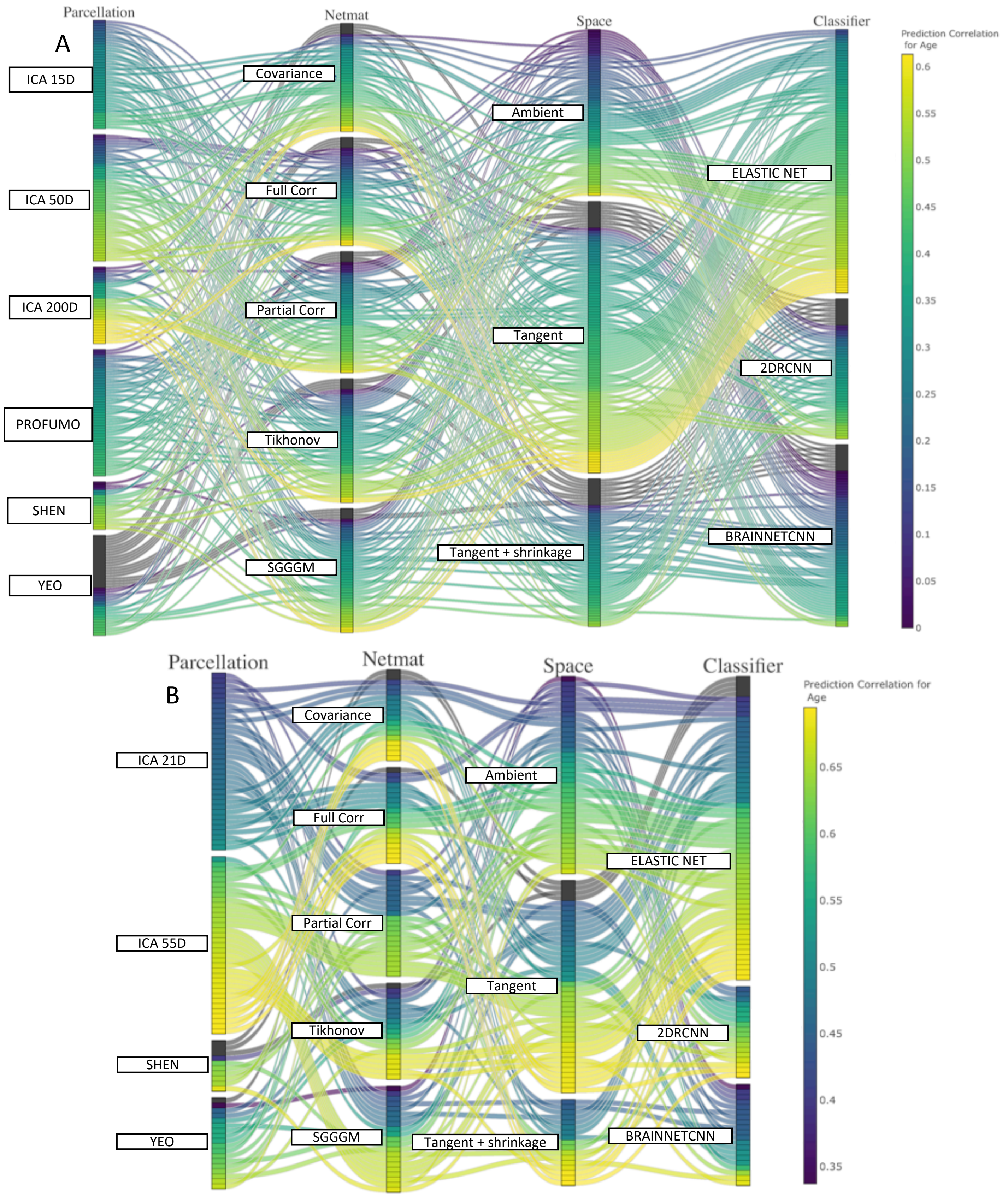

Figure A.26: This parallel coordinates plot provides a visualization of all possible combinations of options in the pipeline to pipeline to predict age from functional connectivity **without deconfounding**. **[A] (HCP Data), [B] (UKB Data):** The lines are color-coded according to their prediction performance.

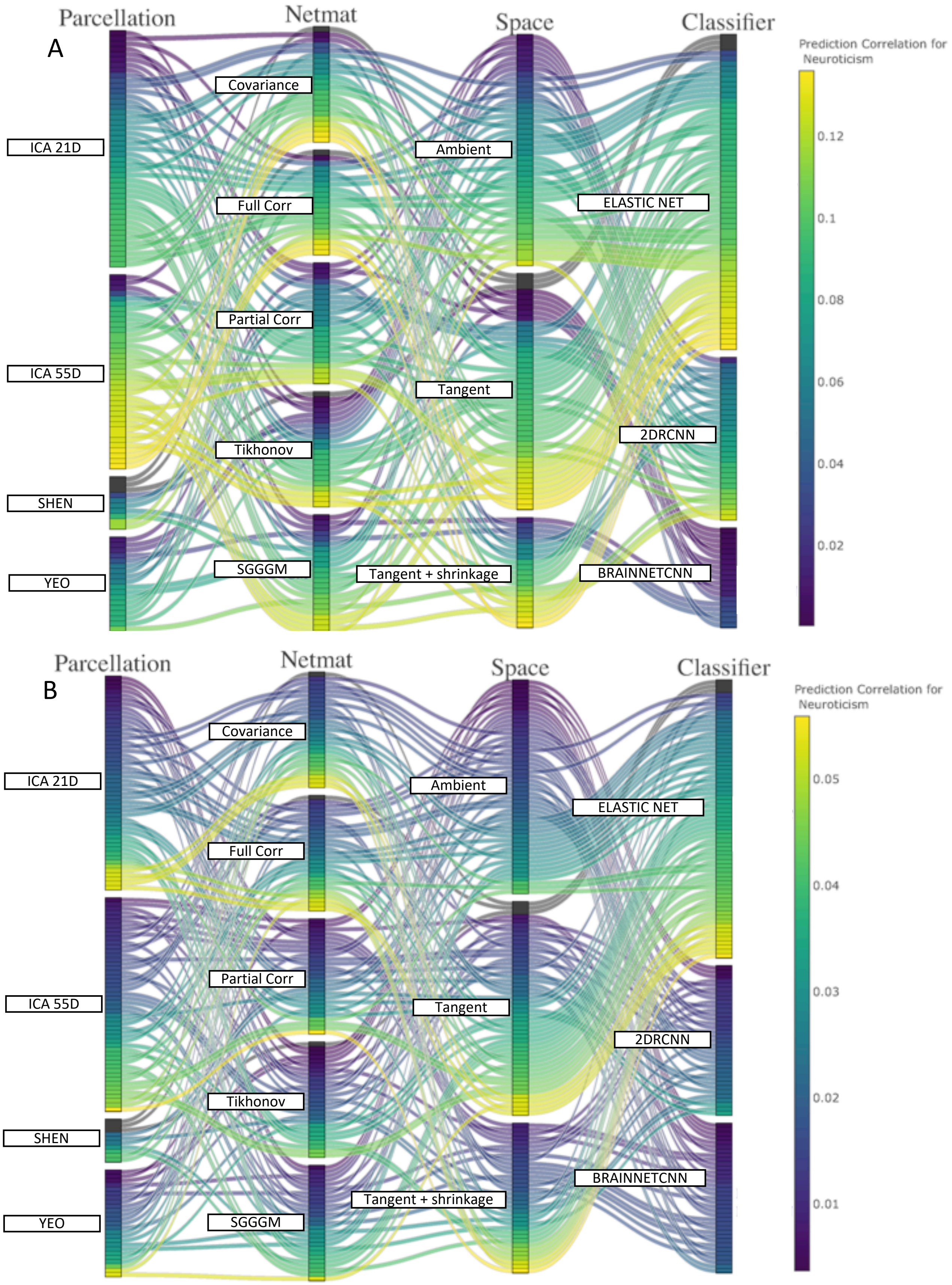

Figure A.27: This parallel coordinates plot provides a visualization of all possible combinations of options in the pipeline to predict neuroticism score from functional connectivity. [**A**] shows the result before confounds removal and [**B**] shows the result after regressing out the confounds.

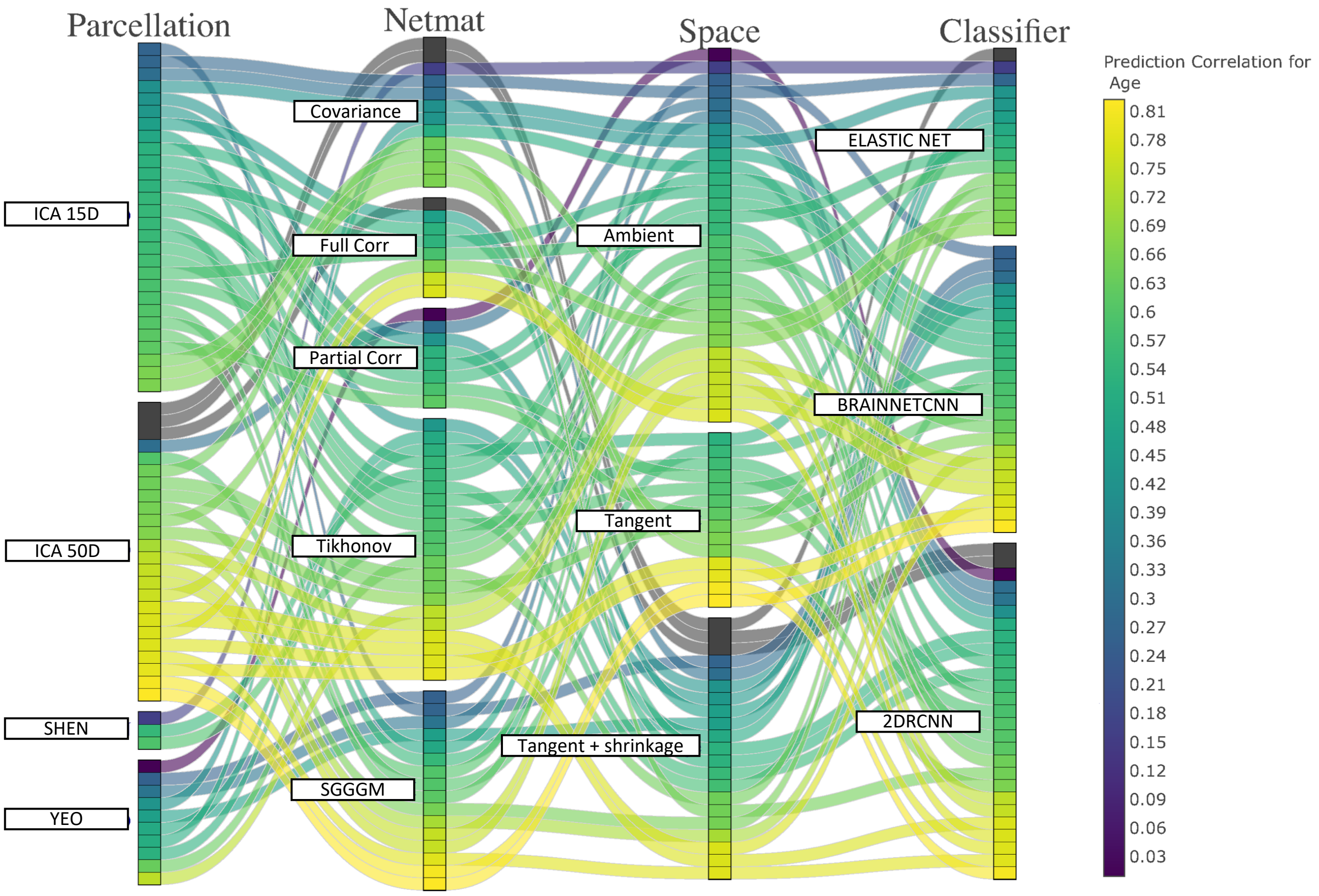

Figure A.28: **(ABIDE)** This parallel coordinates plot provides a visualization of all possible combinations of options in the pipeline to predict age score from functional connectivity. The lines are color-coded according to their prediction performance.

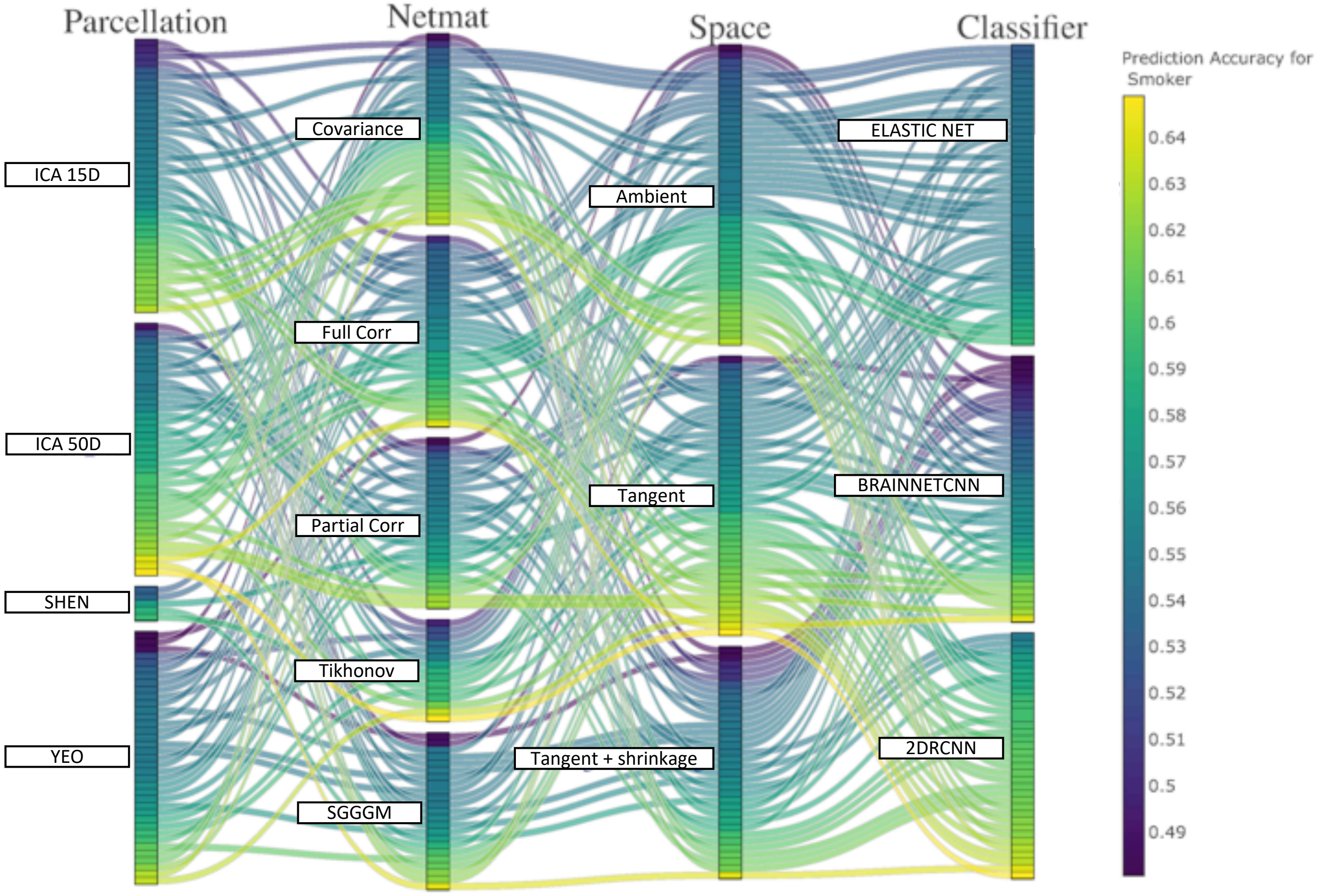

Figure A.29: **(ACPI)** This parallel coordinates plot provides a visualization of all possible combinations of options in the pipeline to predict smoking status from functional connectivity. The lines are color-coded according to their prediction performance.

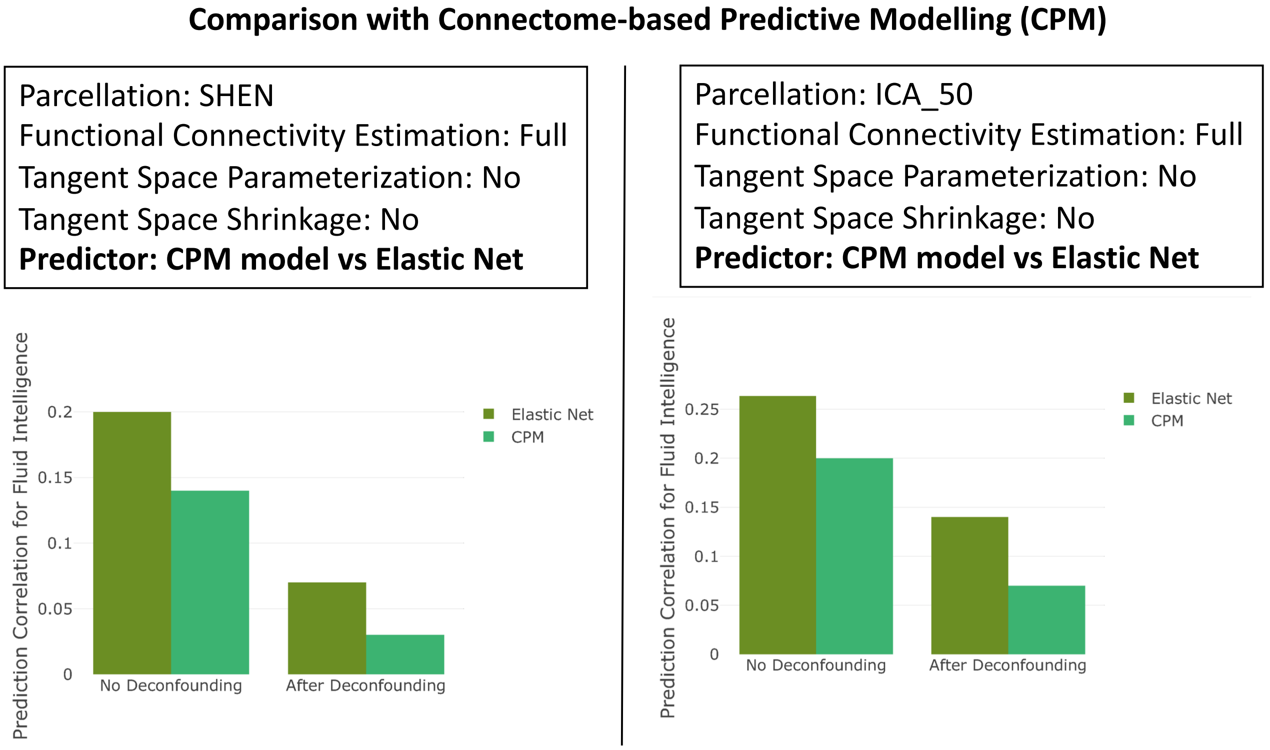

Figure A.30: The comparison of Elastic Net and CPM prediction performance. CPM predictor/classifier is based on a model that averages connectivity edges from a subset of all edges.

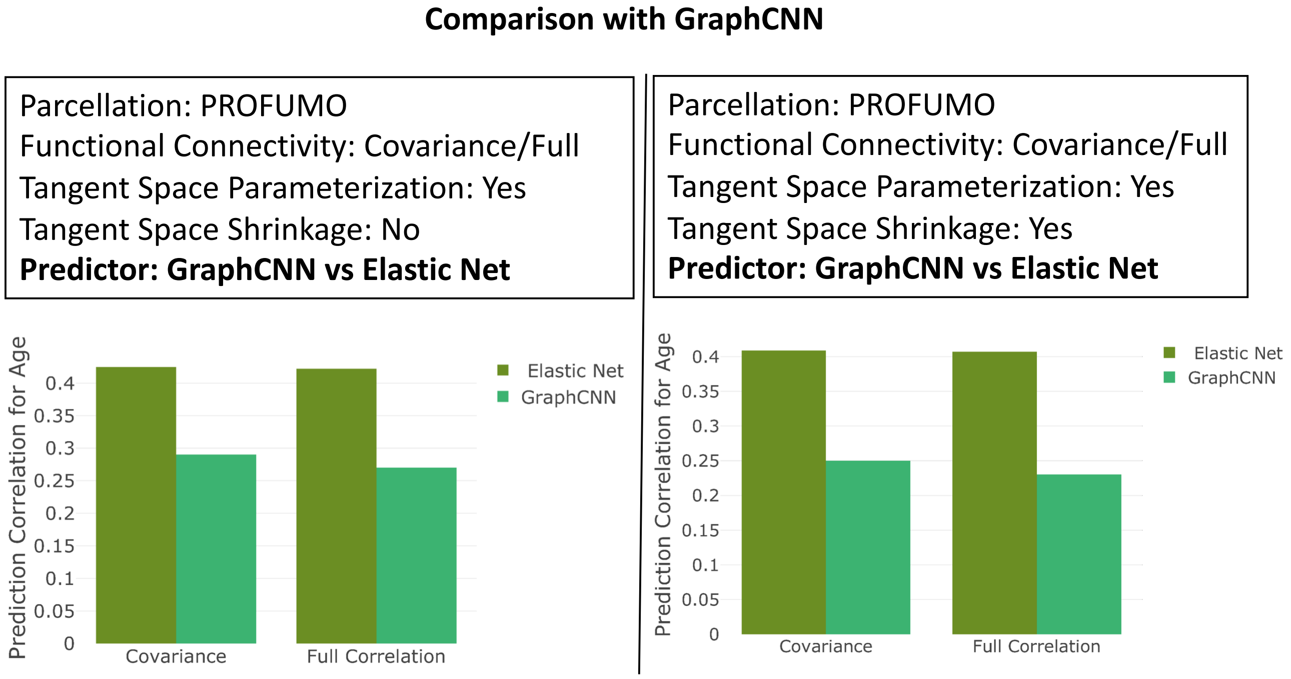

Figure A.31: **(HCP)** The comparison of Elastic Net and GraphCNN prediction performance. We have chosen a subset (e.g., parcellation (PROFUMO), functional connectivity estimation (Full/Covariance)) from all available configurations to illustrate the performance of GraphCNN.

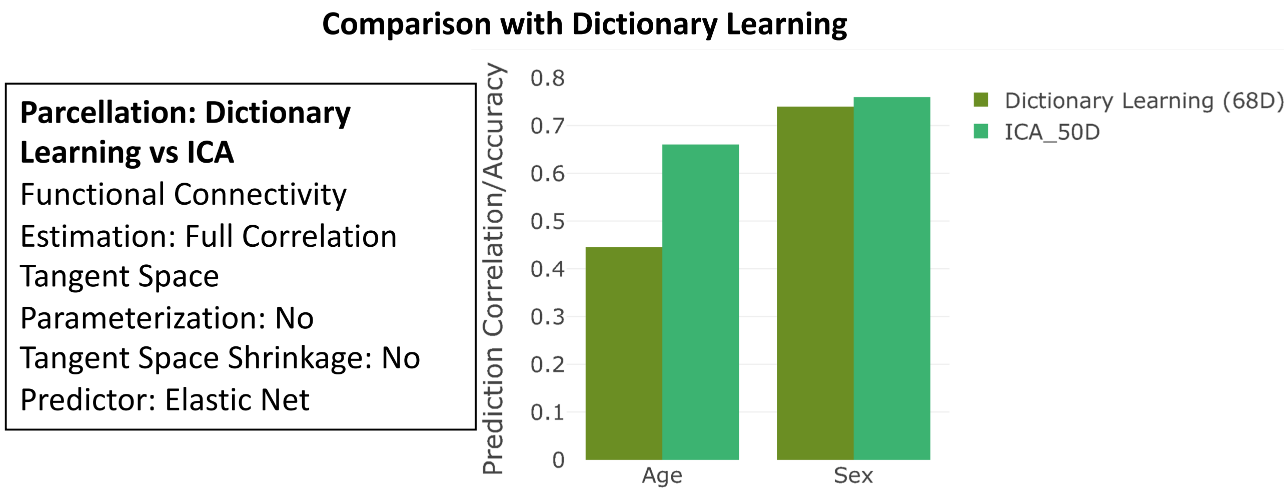

Figure A.32: **(ABIDE)** The comparison of ICA and Dictionary Learning prediction performance. We have chosen a subset (e.g., parcellation (ICA and Dictionary Learning), functional connectivity estimation (Full) from all available configurations to illustrate the comparison.

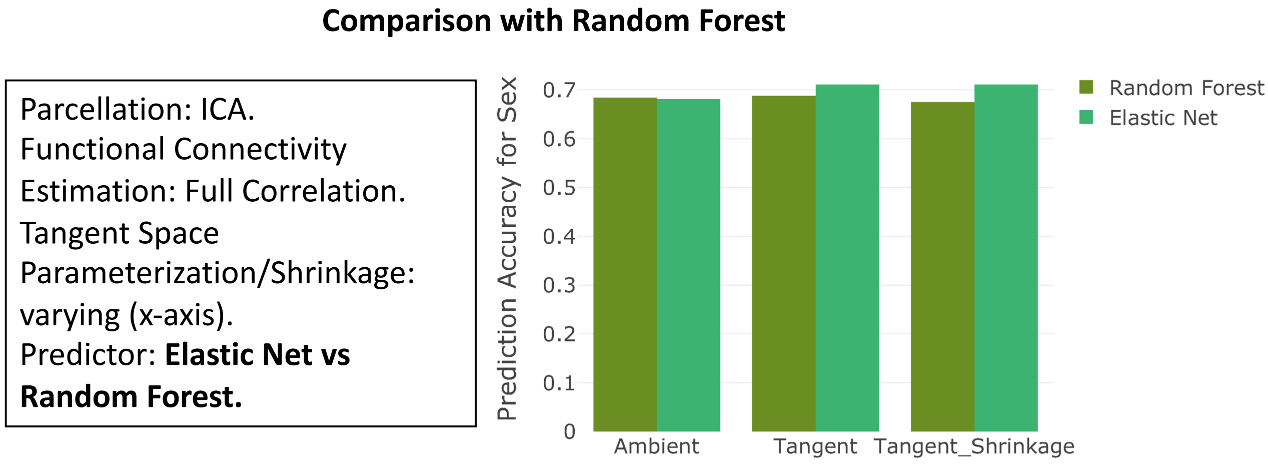

Figure A.33: **(HCP)** The comparison of Elastic Net and Random forest prediction performance. We have chosen a subset (e.g., parcellation (ICA), functional connectivity estimation (Full)) from all available configurations to illustrate the comparison.

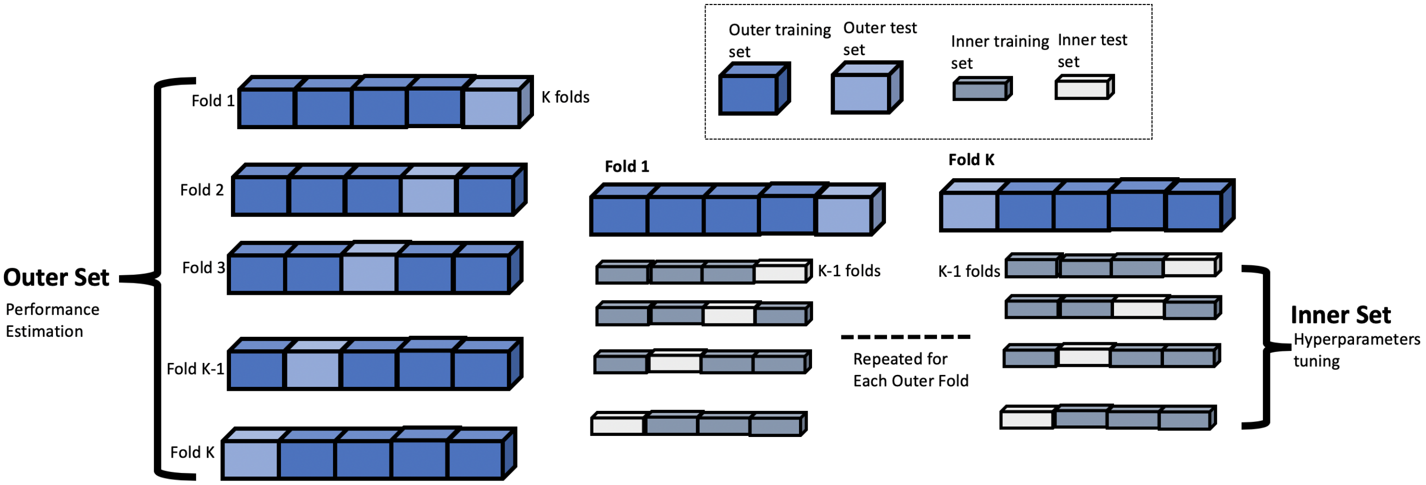

Figure A.34: A summary figure explaining the process of nested cross-validation.
